# Anti-inflammatory and antinociceptive activities of *Solenostemon monostachyus* aerial part extract in mice

**Published:** 2016

**Authors:** Jude Fiom Okokon, Koofreh Davis, Lucky Legbosi Nwidu

**Affiliations:** 1 *Department of Pharmacology and Toxicology, Faculty of Pharmacy, University of Uyo, Uyo, Nigeria *; 2 *Department of Physiology, Faculty of Basic Medical Sciences, University of Uyo, Uyo, Nigeria*; 3 *Department of Pharmacology and Toxicology, Faculty of Pharmacy, Niger Delta University, Wilberforce Island, Bayelsa State*

**Keywords:** *Solenostemon monostachyus*, *Anti-inflammatory*, *Analgesic*

## Abstract

**Objective:**

*Solenostemon monostachyus* is used in traditional medicine for the treatment of various ailments such as ulcer, hypertension, pains and inflammatory diseases. Evaluation of anti-inflammatory and analgesic activities of *S. monostachyus *aerial parts was carried out to ascertain its uses in traditional medicine.

**Materials and Methods:**

The aerial parts of *S. monostachyus* was cold extracted by soaking the dried powdered material in ethanol. The aerial parts crude extract (75 –225 mg/kg) of *S. monostachyus* was investigated for analgesic and anti-inflammatory activities using various experimental models; acetic acid, formalin and thermal- induced pains models for analgesic study and carrageenin, egg albumin and xylene – induced edema models for anti-inflammatory investigation.

**Results:**

The extract caused a significant (p<0.05 – 0.001) dose-dependent reduction of inflammation and pains induced by different phlogistic agents used. These effects were comparable to those of the standard drug, (ASA, 100 mg/kg) used in some models.

**Conclusion:**

The anti-inflammatory and analgesic effects of this plant may in part be mediated through the chemical constituents of the plant and the results of the analgesic action suggest central and peripheral mechanisms. The findings of this work confirm the ethno medical use of this plant to treat inflammatory conditions.

## Introduction

*Solenostemon monostachyus* P. Beauv (Lamiaceae family) is an important herb that is widespread in West and Central Africa. It occurs as an annual weed in anthropogenic habitats and rocky savannahs. It is slightly succulent, aromatic and grows up to 100 cm tall (Mba and Menut, 1994 ▶). The aerial parts of the plant are used in various decoctions traditionally by the Ibibios of the Niger Delta of Nigeria to treat stomach ulcer, fever/malaria (Ajibesin et al., 2008 ▶; Adebayo and Krettli, 2011 ▶), hemorrhoid and other inflammatory diseases. The decoction of the plant is also used as a diuretic as well as to treat hypertension (Koffi et al., 2009 ▶). Phytochemical studies on *S. monostachyus* leaves have revealed the presence of water, proteins, lipids, glucids, calcium, phosphate (Buisson et al., 1965 ▶), essential oil (Mve-Mba et al., 1994 ▶) and phytoconstituents such as diterpenoids (Toshio et al. 1980 ▶), flavonoids, coumarin and polyphenol (Datte et al., 2010 ▶; N’guessan et al., 2011 ▶). The leaf essential oil of S. monostachyus has been reported to contain; β-pinene, oct-1-en-3-ol, β-caryophyllene, octan-3-ol and (E,E)-α-farnesene (Mvé-Mba et al., 1994 ▶). Reported biological activities of the plant include; antioxidant (Datte et al., 2010 ▶; N’guessan et al., 2011 ▶; Okoko and Ere, 2012 ▶), antihypertensive (Fidele et al., 2012 ▶), antimicrobial activities (Ekundayo and Ezeogu, 2006 ▶) and antiulcer (Amazu et al., 2015 ▶). We have reported the analgesic and anti-inflammatory activities of *S. monostachyus* to provide scientific basis for it use in traditional medicine to treat inflammatory diseases. 

## Materials and Methods

**Plants collection**

The plant material *S. monostachyus* (aerial parts) was collected in a farmland in Uyo area, Akwa Ibom State, Nigeria in August, 2014. The plant was identified and authenticated by Dr. Margaret Bassey of Department of Botany and Ecological Studies, University of Uyo, Uyo, Nigeria. Hebarium specimen (FPUU 573) was deposited at Department of Pharmacognosy and Natural Medicine Herbarium.

**Extraction**

The aerial plant parts were washed and shade-dried for two weeks. The dried plants’ materials were reduced to powder using mortar and pistle. Then, the powdered material was soaked in 50% ethanol. The liquid filtrate was concentrated and evaporated to dryness in vacuo 40 C using rotary evaporator and stored in a refrigerator at - 4 C. 

**Phytochemical Screening**

Phytochemical screening of the crude leaf extract was carried out employing standard procedures and tests (Trease and Evans, 1989 ▶, Sofowora, 1993 ▶), to reveal the presence of chemical constituents such as alkaloids, flavonoids, tannins, terpenes, saponins, anthraquinones, cardiac glycosides among others.

**Animals**

Albino Swiss mice (17 – 25g) of either sex were obtained from the University of Uyo animal house. They were maintained on standard animal pellets and water ad libitum. Permission and approval for animal studies were obtained from the College of Health Sciences Animal Ethics committee, University of Uyo.

**Determination of median lethal dose (LD50)**

The median lethal dose (LD_50_) of the extract was estimated using albino mice by intraperitoneal (i.p) route using the method of Miller and Tainter (1944) ▶. This involved intraperitoneal administration of different doses of the extract (100 – 1000 mg/kg) to groups of five mice each. The animals were observed for manifestation of physical signs of toxicity such as writhing, decreased motor activity, decreased body/limb tone, decreased respiration and death. The number of deaths in each group within 24 hours was recorded. 

**Evaluation of anti-inflammatory activity of the extract**

*Carrageenin – induced mice hind paw oedema*

Adult albino male mice were used after a 24-hour fast and deprived of water only during experiment. Inflammation of the hind paw was induced by injection of 0.1 ml of freshly prepared carrageenin suspension in normal saline into the sub planar surface of the hind paw. The linear circumference of the injected paw was measured before and 0.5, 1, 2, 3, 4 and 5 hours after the administration of phlogistic agent. The increase in paw circumference in post administration of phlogistic agent was adopted as the parameter for measuring inflammation (Winter et al., 1962 ▶; Akah and Nwambie, 1994 ▶; Ekpendu et al., 1994 ▶, Besra et al., 1996 ▶; Nwafor et al., 2010 ▶). The difference in paw circumference between the control and 0.5, 1, 2, 3, 4 and 5 hrs after the administration of phlogistic agent was used to assess the inflammation (Hess and Milonig, 1992 ▶). The extract (75, 150 and 225 mg/kg i.p) was administered to various groups of 6 mice each, 1 h before inducing inflammation. Control mice received carrageenin while reference group received ASA (100 mg/kg). The average (mean) edema was assessed by measuring with Vernier calipers.

**Egg-albumin induced inflammation**

Inflammation was induced in mice by the injection of egg albumin (0.1ml, 1% in normal saline) into the sub planar tissue of the right hind paw (Akah and Nwambie, 1994 ▶; Okokon and Nwafor, 2010 ▶). The linear circumference of the injected paw was measured before and 0.5, 1, 2, 3, 4 and 5 hrs after the administration of the phlogistic agent. The extract (75, 150 and 225 mg/kg i.p) and ASA (100 mg/kg orally) were administered to groups (n=6) of 24 h fasted mice 1 h before the induction of inflammation. The control group received 10 ml/kg of distilled water orally. Edema (inflammation) was assessed as the difference in paw circumference between the control and 0.5, 1, 2, 3, 4 and 5 hrs post administration of the phlogistic agent (Hess and Milonig, 1972 ▶). The average (mean) edema was assessed by measuring with vernier calipers.

**Xylene – induced ear oedema**

Inflammation was induced in mice by topical administration of 2 drops of xylene at the inner surface of the right ear. The xylene was left to act for 15 mins. *S. monostachyus* extract (75, 150 and 225 mg/kg i.p), dexamethasone (4 mg/kg) and distilled water (0.2 ml/kg) were orally administered to various groups (n=6) of mice 1 h before the induction of inflammation. The animals were sacrificed under light anaesthesia and the left ears cut off. The difference between the ear weights was taken as the oedema induced by the xylene (Tjolsen et al., 1992 ▶; Mbagwu et al., 2007 ▶; Okokon and Nwafor, 2010 ▶).

**Evaluation of analgesic potential of the extract**

*Acetic acid induced writhing in mice*

Writhings (abdominal constrictions consisting of the contraction of abdominal muscles together with the stretching of hindlimbs) resulting from intraperitoneal (i.p) injection of 2% acetic acid, was induced according to the procedure described by Santos et al. (1994) ▶, Correa et al. (1996) ▶ and Nwafor et al., (2010) ▶. The animals were divided into 5 groups of 6 mice per group. Group 1 served as the negative control and received 10 ml/kg of normal saline, while groups 2, 3 and 4 were pre-treated with 75, 150, and 225 mg/kg doses of *S. monostachyus* extract intraperitoneally, and group 5 received 100 mg/kg of acetyl salicylic acid. After 30 minutes, 0.2 ml of 2% acetic acid was administered intraperitoneally (i.p). The number of writhing movements was counted for 30 minutes. Antinociception (analgesia) was expressed as the reduction of the number of abdominal constrictions between the control animals and mice pretreated with extracts.

**Formalin – induced hind paw licking in mice**

The procedure adopted was similar to that described by Hunskaar and Hole (1987) ▶, Correa and Calixto (1993) ▶, Gorki et al., (1993) ▶ and Okokon and Nwafor, (2010) ▶. The animals were injected with 20 μL of 2.5% formalin solution (0.9% formaldehyde) made up in phosphate buffer solution (PBS concentration: Nacl 137 mM, Kcl 2.7 mM and phosphate buffer, 10 mM) under the surface of the right hind paw. The amount of time spent licking the injected paw was timed and considered as the indication of pain. Adult albino mice (20 – 25 g) of either sex randomized into five groups of 6 mice each were used for the experiment. The mice were fasted for 24 hours before being used but allowed access to water. The animals in group 1 (negative control) received 10 ml/kg of normal saline, groups 2 - 4 received 75, 150, and 225 mg/kg doses of the extract, while group 5 received 100 mg/kg of acetyl salicylic acid (ASA) 30 minutes before being challenged with buffered formalin. The responses were measured for 30 mins after formalin injection. 

**Thermally induced pain in mice**

The effect of extract on hot plate induced pain was investigated in adult mice. The hot plate was used to measure the response latencies according to the method of Vaz et al. (1996) ▶ and Okokon and Nwafor, (2010). ▶ In these experiments, the hot plate was maintained at 45±1oC, each animal was placed into a glass beaker of 50 cm diameter on the heated surface, and the time(s) between placement and shaking or licking of the paws or jumping was recorded as the index of response latency. An automatic 30-second cut-off was used to prevent tissue damage. The animals were randomly divided into 5 groups of 6 mice each and fasted for 24 hours but allowed access to water. Animalgroup 1 served as negative control and received 10 ml/kg of normal saline. Groups 2, 3 and 4 were pre-treated intraperitoneally with 75, 150, and 225 mg/kg doses of *S. monostachyus* extract respectively, while animal group 5 received 100 mg/kg of acetyl salicylic acid intraperitoneally, 30 minutes prior to the placement on the hot plate. 

**Statistical analysis and data evaluation**

 Data obtained from this work were analyzed statistically using ANOVA (One- way) followed by a post test (Tukey-Kramer multiple comparison test). Differences between the means were considered significant at 1% and 5% level of significance i.e. p < 0.01and 0.05.

## Results

**Phytochemical screening**

The phytochemical screening of the ethanol aerial extract of *S. monostachyus* revealed the presence of alkaloids, cardiac glycosides, flavonoids. saponins, tannins and terpenes. 

**Determination of Median lethal dose (LD50)**

The median lethal dose (LD_50_) was calculated to be 766.35±37.56 mg/kg. The physical signs of toxicity included excitation paw licking, increased respiratory rate, decreased motor activity, gasping and coma which was followed by death.

**Carragenin-induced oedema in mice**

The effect of ethanolic extract *of S. monostachyus* on carragenin-induced oedema is as shown in Table 1. The extract (75 - 225 mg/kg) exerted a significant (p<0.05 – 0.001) anti-inflammatory effect in a dose –dependent manner which was comparable to the standard drug, ASA, 100 mg/kg (Table 1a and 1b). 

**Table 1a:** Effect of *Solenostemon monostachyus* extract on carrageenin- induced edema in mice

**Treatment/** **Dose (mg/kg)**	**Time intervals (hr)**						
	**0**	**0.5**	**1**	**2**	**3**	**4**	**5**
**Control**	3.14 0.21	7.44 0.22	7.72 0.21	7.54 0.38	6.40 0.12	6.28 0.14	5.82 0.10
**Extract** **75**	3.22 0.17	5.79 0.26	6.86 0.10	5.56 0.31	5.17 0.08	4.66 0.05 ^	4.02 0.03 ***
**150**	3.11 0.10	5.88 0.15	6.42 0.12*	5.43 0.10***	5.04 0.01***	4.44 0.01 ***	3.74 0.03 ***
**225**	3.10 0.01	5.91 0.31	6.29 0.03*	5.27 0.29***	4.63 0.08***	4.23 0.05***	3.57 0.10 ***
**ASA 100**	3.26 0.11	5.610.31*	6.40 0.35*	5.81 0.43***	4.57 0.54***	4.28 0.22***	3.67 0.11***

Data are expressed as mean SEM. Significant at

* p<0.05;

** p< 0.01,

*** p< 0.001 when compared to control. n = 6.

**Table 1b:** Effect of *Solenostemon monostachyus* extract on carrageenin induced oedema in mice

**Treatment/** **Dose (mg/kg)**	**Average inflammation (mm) ± SEM**					
	**0.5hr**	**1hr**	**2hr**	**3hr**	**4hr**	**5hr**
**Control**	4.30 0.22	4.58 0.21	4.40 0.38	3.26 0.12	3.14 0.14	2.68 0.10
**Extract** **75**	2.57 0.26	3.04 0.10***	2.34 0.31***	1.95 0.08***	1.40 0.07***	0.80 0.05 ***
**150**	2.77 0.15	3.31 0.12**	2.32 0.10***	1.93 0.01***	1.33 0.01***	0..630.05 ***
**225**	2.81 0.31	3.19 0.03***	2.17 0.29***	1.53 0.08***	1.13 0.05***	0.47 0.10 ***
**ASA 100**	2.350.31*	3.14 0.35***	2.55 0.43***	1.31 0.54***	1.03 0.22***	0.41 0.11***

Data are expressed as mean SEM. Significant at

** p< 0.01,

*** p< 0.001 when compared to control. n = 6

**Egg albumin- induced edema**

Administration of the extract of *S. monostachyus* (75-225 mg/kg) on egg albumin - induced edema in mice caused a significant (p<0.05-0.001) dose-dependent anti-inflammatory effect against edema caused by egg albumin. The effect was comparable to that of the standard drug, ASA (100 mg/kg) (Table 2a and 2b).

**Xylene- induced ear edema**

The anti-inflammatory effect of crude extract of *S. monostachyus* against xylene-induced ear edema in mice is shown in Table 3. The extract exerted a dose-dependent anti-inflammatory effect which was only significant (p<0.01) at the highest dose (225 mg/kg) but incomparable to that of the standard drug, dexamethasone (4.0 mg/kg).

**Table 2a:** Effect of *Solenostemon monostachyus *extract on egg- albumin induced edema in mice

**Treatment/** **Dose (mg/kg)**	**Time intervals (hr)**						
	**0**	**0.5**	**1**	**2**	**3**	**4**	**5**
**Control**	2.65 0.07	3.35 0.25	3.27 0.06	3.15 0.11	3.06 0.07	2.95 0.07	2.90 0.06
**Extract** **75**	2.56 0.01	3.19 0.16	3.08 0.02	2.86 0.06	2.72 0.05	2.88 0.02	2.77 0.01
**150**	2.77 0.05	3.16 0.11	2.90 0.06^*^	2.82 0.09^***^	2.85 0.07^***^	2.62 0.04^***^	2.59 0.04^ ***^
**225**	2.60 0.04	3.15 0.04	2.88 0.06^*^	2.78 0.06^***^	2.68 0.03^***^	2.67 0.04^***^	2.62 0.04^ ***^
**ASA 100**	2.61 0.06	2.99 0.16^*^	2.89 0.02^*^	2.84 0.02^***^	2.76 0.03^***^	2.64 0.05^***^	2.64 0.05^***^

Data are expressed as mean SEM. Significant at

* p<0.05;

** p< 0.01,

*** p< 0.001 when compared to control. n = 6.

**Table 2b:** Effect of *Solenostemon monostachyus *extract on egg- albumin induced oedema in mice

**Treatment/** **Dose (mg/kg)**	**Average inflammation (mm) ± SEM**					
	**0.5hr**	**1hr**	**2hr**	**3hr**	**4hr**	**5hr**
**Control**	0.70 0.01	0.62 0.01	0.50 0.01	0.41 0.01	0.30 0.14	0.25 0.10
**Extract** **75**	0.63 0.0.2	0.52 0.01	0.30 0.01***	0.16 0.01***	0.11 0.01 ***	0.01 0.01***
**150**	0.330.01***	0.13 0.01***	0.14 0.01***	0.08 0.01***	0.06 0.01 ***	0.00 0.00***
**225**	0.550.01***	0.28 0.01***	0.18 0.01***	0.08 0.01***	0.07 0.01***	0.02 0.01 ***
**ASA 100**	0.380.01***	0.28 0.01***	0.23 0.01***	0.15 0.01***	0.03 0.01***	0.03 0.01***

Data are expressed as mean SEM. Significant at

*** p < 0.001 when compared to control. n = 6

**Table 3:** Effect of *Solenostemon monostachyus *extract on xylene-induced ear oedema in mice.

**Treatment/** **Dose (mg/kg)**	**Weight of the right ear (g)**	**Weight of the left ear (g)**	**Increase in ear weight (g)**	**% inhibition**
**Control (normal saline) 0.2ml**	0.080 0.01	0.033 0.00	(139.39) 0.046 0.00	
**Extract** **75**	0.076 0.01	0.036 0.00	(111.11) 0.040 0.00^ NS^	13.04
**150**	0.088 0.01	0.033 0.01	(100.0) 0.033 0.01^ NS^	28.26
**225**	0.063 0.01	0.036 0.01	(72.22) 0.026 0.01*	43.47
**Dexamethasone 4.0**	0.043 0.01	0.036 0.01	(27.77) 0.01 0.00**	97.82

Figures in parenthesis indicate % increase in ear weight.

* Significant at p<0.01,

** p<0.001 when compared with control. n = 6

**Effect of ethanol crude extract of** ***S. monostachyus*** **on acetic acid-induced writhing in mice**

The administration of *S. monostachyus *extract (75 - 225 mg/kg) demonstrated a dose-dependent reduction in acetic acid-induced writhing in mice. The reductions were statistically significant (p<0.05- 0.001) relative to control and incomparable to that of the standard drug, ASA (Table 4).

**Table 4:** Effect of *Solenostemon monostachyus* extract on acetic acid induced writhing in mice

**Treatment/** **Dose(mg/kg)**	**Time intervals (hr)**						
	**5**	**10**	**15**	**20**	**25**	**30**	**Total**
**Control**	3.33 0.56	9.00 1.00	16.00 1.30	16.66 1.76	13.71 2.66	8.76 0.65	65.46 7.93
**Extract** **75**	5.03 0.57	5.35 1.20	15.34 1.52	16.0 1.00	11.00 1.20	8.33 0.62	53.05 6.11*
**159**	4.00 0.57	3.34 0.20 **	11.00 1.15*	9.46 0.33*	6.60 0.56***	4.33 0.31***	38.73 3.12***
**225**	0.000.00***	1.00 0.57 ***	8.00 1.00***	7.33 0.30***	6.33 1.15***	3.00 0.00***	25.66 3.02***
**ASA 100**	1.73 0.20*	3.33 0.66**	4.43 0.88***	5.00 0.28***	0.34 0.23***	1.66 0.66**	16.49 2.91***

Data are expressed as meanSEM. Significant at

* p< 0.05,

** p< 0.01,

*** p< 0.001 when compared to control n = 6.

**Effect of ethanol extract of** ***S. monostachyus*** **on formalin-induced hind paw licking in mice**

The extract exhibited a non dose- dependent analgesic effect on formalin-induced hind paw licking in mice. The extract prominently inhibited the two phases of formalin-induced pains with a more considerable inhibition of the second phase. These inhibitions were significant relative to the control (p< 0.05-0.001) and comparable to that of the standard drug, ASA (Table 5).

**Effect of ethanolic crude extract of** ***S. monostachyus*** **on thermally-induced pain in mice**

The extract (75 - 225 mg/kg) exhibited a dose - dependent effect on thermally-induced pain in mice. This inhibition was only statistically significant (p<0.001) relative to the control at the highest dose of the extract (225 mg/kg) (Table 6)**.**

**Table 5:** Effect of *Solenostemon monostachyus* extract on formalin–induced hind paw licking in mice

**Treatment/** **Dose (mg/kg)**	**Time intervals (mins)**						
	**5**	**10**	**15**	**20**	**25**	**30**	**Total**
**Control**	14.00 1.15	8.02 1.00	8.33 0.57	8.00 0.57	9.00 0.87	14.00 2.08	61.35 6.24
**Extract** **75**	9.00 0.01	2.66 0.33***	2.66 0.31***	2.66 0.58***	1.66 0.67***	0.00 0.00***	18.64 1.90***
**150**	14.64 1.40	0.00 0.00***	0.00 0.00***	0.66 0.66***	2.00 0.16***	1.33 0.33***	18.63 2.55***
**225**	6.66 0.66	0.00 0.00***	1.33 0.00***	0.33 0.33***	3.33 0.32***	0.66 0.66***	12.31 1.97***
**ASA 100**	7.66 0.51	3.00 0.00***	2.33 0.85***	2.66 0.45***	3.33 0.66***	3.00 0.37***	21.98 2.84***

Data are expressed as mean SEM. Significant at

*** p < 0.001. When compared to control ,n = 6.

**Table 6:** Effect of *Solenostemon monostachyus* extract on the hot plate test

**Group**	**Dose** **Mg/kg**	**Reaction time (sec)** **(mean SEM)**	**% inhibition**
**Control**	-	3.53 0.51	
***S.monostachyus***	75	3.96 0.31	12.18
	150	4.94 0.37	39.94
	225	5.50 0.64**	55.80
**ASA**	100	11.15 0.36***	215.86

Data are expressed as mean SEM. Significant at

** p< 0.05,

*** p< 0.001 when compared to control, n = 6.

## Discussion

*S. monostachyus* is used traditionally for the treatment of various illnesses such as fever, pains and inflammatory conditions. In this study, the ethanol aerial parts extract was evaluated for anti-inflammatory and analgesic activities using various experimental models.

In the carragenin-induced oedema, the extract (75 - 225 mg/kg) was observed to have exerted significant effect at the early stage of inflammation (1-2 hr) indicating an effect probably on histamine, serotonin and kinnins that are involved in the early stage of carragenin-induced oedema (Vane and Booting, 1987 ▶). The extract further reduction of the later stage of the oedema may be due to its ability to inhibit prostaglandin which is known to mediate the second phase of carragenin induced inflammation (Vane and Booting, 1987 ▶). Similarly, ASA (100 mg/kg) whose mechanism of action involves inhibition of prostaglandin, produced a considerable inhibition of the paw swelling induced by carragenin injection.

The extract also inhibited egg albumin-induced oedema demonstrating that it can inhibit inflammation by blocking the release of histamine and 5-HT, two mediators that are released by egg albumin (Nwafor et al., 2007 ▶). However, ASA, a cyclooxygenase inhibitor, significantly reduced oedema produced by egg albumin.

The extract exerted a significant (P<0.01) inhibition of ear oedema caused by xylene only at the highest dose of the extract, suggesting the inhibition of phospholipase A_2_ which is involved in the pathophysiology of inflammation due to xylene (Lin et al.,1992 ▶). However, dexamethasone, a steroid antiinflammatory agent produced significant reduction in the mean right ear weight of the positive control rats indicating an inhibition of PLA_2_.

The extract significantly reduced acetic acid-induced writhing, formalin-induced hind paw licking and also delayed the reaction time of animals (mice) to thermally induced pain. Acetic acid causes inflammatory pain by inducing capillary permeability (Amico-Roxas et al.,1984 ▶; Nwafor et al., 2007 ▶), and in part through local peritoneal receptors from peritoneal fluid concentration of PGE_2 _and PGF_2_α (Deraedt et al.,1980 ▶; Bentley et al.,1983 ▶). The acetic acid-induced abdominal writhing is a visceral pain model in which the processor releases arachidonic acid via cyclooxygenase, and prostaglandin biosynthesis plays a role in the nociceptive mechanism (Franzotti et al., 2002 ▶). It is used to distinguish between central and peripheral pain. These results suggest that the extract may be exerting its action partly through the lipoxygenase and/or cyclooxygenase system. 

The inhibition of acetic acid-induced writhing by the extract at all of the doses suggests an antinociceptive effect which might have resulted from the inhibition of the synthesis of arachidonic acid metabolites.

Formalin- induced pains involve two different types of pains which are in phases; neurogenic and inflammatory pains (Vaz et al., 1996 ▶, 1997) and measure both centrally and peripherally mediated activities that are characteristic of biphasic pain responses. The first phase (0 to 5 min), named neurogenic phase provoked the release of bradykinin and substance P while the second and late phase initiated after 15 to 30 min of formalin injection resulted in the release of inflammatory mediators such as histamine and prostaglandin (Wibool et al., 2008 ▶; Yi-Yu et al., 2008 ▶). The first phase of formalin-induced hind paw licking is selective for centrally acting analgesics such as morphine (Berken et al.,1991 ▶), while the late phase of formalin-induced hind paw licking is peripherally mediated. The extract ability to inhibit both phases of formalin-induced paw licking suggests its central and peripheral activities as well as its ability to inhibit bradykinins, substance P, histamine and prostaglandins which are mediators in these pains.

The study also shows that the extract significantly delayed the reaction time of the thermally- induced (hot plate) test. This model is selective for centrally acting analgesics and indicates narcotic involvement (Turner, 1995 ▶) with opioid receptors. 

Some phytochemical constituents such as diterpenoids (Toshio et al. 1980 ▶), flavonoids, coumarin, polyphenol (Datte et al., 2010 ▶; N’guessan Hugues et al., 2011 ▶) as well as β-pinene, oct-1-en-3-ol, β-caryophyllene, octan-3-ol and (*E,E*)-α-farnesene (Mvé-Mba et al., 1994 ▶) have been reported to be present in the leaf extract of *S. monostachyus*. Some of these phytoconstituents have been found to be present in the aerial extract of plant in this study and maybe responsible for the observed reported activities in this study.

β-pinene, a monoterpene, has been reported to possess analgesic (Liapi et al., 2007) and anti-inflammatory activity (Lorente et al., 1989), while the sesquiterpenes present (β-caryophyllene and (E,E)-α-farnesene) have been reported to possess analgesic and anti-inflammatory potentials (Ahmed et al., 1997 ▶; Datta et al., 2004 ▶; Chavan et al., 2012 ▶) and may also contribute to the observed anti-inflammatory and analgesic activities.

Flavonoids are known as anti-inflammatory compounds acting through inhibition of the cyclooxygenase and lipoxygenase pathways (Liang et al., 1999 ▶; Carlo et al., 1999 ▶), phospholipase A_2_ and phospholipase C (Middleton et al., 2000). Some flavonoids exert their antinociception via opioid receptor activation activity (Suh et al., 1996 ▶; Rajendran et al., 2000 ▶; Otuki et al., 2005 ▶). 

The extract above has been reported to exhibit anti-inflammatory and analgesic activities. The presence of these compounds (polyphenolics, flavonoids, monoterpenes, sesquiterpenes and triterpenes) in this plant might account for these activities and may in part explain the mechanisms of its actions in this study. 

In conclusion, the results of this study demonstrated that *S. monostachyus *possesses anti-inflammatory and analgesic properties. Further investigation is being advocated especially in elucidating cellular mechanisms and establishing structural components of the active ingredients with a view of standardizing them**.**
